# How job demands and job resources contribute to our overall subjective well-being

**DOI:** 10.3389/fpsyg.2023.1220263

**Published:** 2023-07-19

**Authors:** Sara Claes, Sophie Vandepitte, Els Clays, Lieven Annemans

**Affiliations:** Department of Public Health and Primary Care, Ghent University, Ghent, Belgium

**Keywords:** job demands-resources theory, well-being, subjective well-being, leadership, structural equation (SEM)

## Abstract

**Objectives:**

How the work environment contributes to employees’ overall subjective well-being remains inadequately explored. Building upon the seminal Job Demands-Resources model, this study aims to test a complex model that combines leadership, job demands, and job resources, as factors contributing either indirectly (via job satisfaction) or directly to employees’ subjective well-being (SWB).

**Methods:**

The cross-sectional data (*N* = 1,859) of the Belgian National happiness study (2020) were used. Leadership (satisfaction with leadership; perceived supervisor support), job demands (role conflict; job insecurity; work-private conflict; perceived working conditions), job resources (autonomy; relatedness; competence; skill utilization; personal growth), job satisfaction and subjective well-being (life evaluation; positive affect; negative affect) were assessed via self-report questionnaires. The proposed model investigates the direct impact of job demands and resources on SWB, as well as the indirect impact with job satisfaction as mediating factor, and was tested using the Structural Equation Modeling technique.

**Results:**

Findings supported the proposed model. Both job demands and job resources have a *direct* relationship with SWB. Job resources are positively related to overall SWB, whereas job demands negatively affected SWB. Moreover, job resources are more strongly related to SWB compared to job demands. The demands and resources also indirectly contribute to employee’s SWB via job satisfaction as job satisfaction appeared to mediate these relationships.

**Conclusion:**

The current study shows that both job demands and resources directly and indirectly contribute to employees’ SWB. Creating a supportive and healthy work environment is thus of paramount importance in order to foster employees’ SWB. In particular, investing in improving job resources may be a fruitful approach to promote employees’ overall subjective well-being.

## Introduction

Professionally active people are generally in better health than their unemployed counterparts (Sandmark, 2011; Kim and von dem Knesebeck, 2015; Vanthomme and Gadeyne, 2019) and tend to be more happy than those who are not professionally active (McKee-Ryan et al., 2005; Dolan et al., 2008; Tay and Harter, 2013). Work thus seem to have the potential to cultivate employees’ health and overall well-being. However, work can also be a burden resulting in several healthcare problems such as burnout and depression (Bonde, 2008; Bakker and de Vries, 2021). How job characteristics and the work environment positively or negatively affect employees’ general subjective well-being (Diener, 1984) remains inadequately explored. Yet, understanding how the job and its characteristics affect the overall well-being of employees is of key importance. Indeed, ensuring the well-being of the workforce matters to both organizations and society as a whole as the costs associated with ill-being are immense (Trautmann et al., 2016).

A seminal theory concerned with employee well-being is the Job Demands-Resources model (Demerouti et al., 2001; Bakker and Demerouti, 2007; Bakker and Demerouti, 2017). According to this framework, two independent processes affect work engagement and wellbeing at work (Demerouti et al., 2001; Schaufeli and Bakker, 2004; Bakker and Demerouti, 2017). On the one hand, high job demands, i.e., the aspects of work that require sustained effort, gradually drain the resources of employees and by doing so contribute to job strain, burnout and ill-health. This is known as the *energy depletion or health-impairment process*. On the other hand, job resources, i.e., the aspects of the job that facilitate goal achievement, stimulate personal growth and development, and lower the job demands and their associated physiological and psychological costs, induce a *motivational process* and as such contribute to employee engagement and other positive attitudes (Schaufeli and Bakker, 2004; Bakker and Demerouti, 2007; Bakker, 2011).

Research on the JD-R model also acknowledges the role of leadership in explaining employee well-being (Tummers and Bakker, 2021; Bakker et al., 2023). In these studies, leadership is often seen as either a job resource (in the case of positive leadership concepts) or a job demand (in the case of negative leadership concepts). Other researchers argue that leadership directly impacts the job demands and job resources as leaders shape the work environment and set the conditions in which employees can flourish or suffer. From this point of view leadership is considered an antecedent of demands and resources (Schaufeli, 2015; Bakker and Demerouti, 2017; Berthelsen et al., 2018; Tummers and Bakker, 2021; Bakker et al., 2023). In this study we will follow the latter conceptualization implying that leadership affects the degree of demands and resources at work.

The literature on the JD-R model is extensive and has well supported its assumptions (Crawford et al., 2010; Alarcon, 2011; Lesener et al., 2019; Galanakis and Tsitouri, 2022; Mazzetti et al., 2023), but is mainly concerned with the impact of the work environment in terms of leadership, job demands and job resources on health and well-being outcomes directly related to work (e.g., work engagement, absence of burnout, job satisfaction etc.). However, in the well-being literature a distinction is made between domain-specific measures of well-being (e.g., work engagement, job satisfaction) and general well-being indicators (e.g., subjective well-being, life satisfaction; Page and Vella-Brodrick, 2009; Stephan et al., 2023). Limiting the well-being of employees to work-related well-being outcomes thus may be too narrow as work is only one aspect of life. As such, the interplay between work and non-work roles has increasingly received scientific attention and research on work-home interference has demonstrated that experiences at work influence family life and vice versa (Ten Brummelhuis and Bakker, 2012; Bakker et al., 2023). Other researchers found evidence for a spill-over between well-being at work and subjective well-being (Bowling et al., 2010; Hakanen and Schaufeli, 2012; Upadyaya et al., 2016; Weziak-Bialowolska et al., 2020). Also, results of a recent meta-analysis demonstrated that work engagement is related to employees’ overall well-being in terms of better health, greater life satisfaction and lower psychological distress (Mazzetti et al., 2023). These findings further suggest that work affects employees’ overall well-being. Notwithstanding the gaining scientific attention for the relation between well-being at work and overall well-being, the impact of the work environment itself (in terms of job demands and job resources) on employees’ subjective well-being is not yet well understood. There are a few studies that investigated the direct relationship between job characteristics and general well-being indicators. The results of a recent study for instance showed that employees’ job demands are negatively related to their overall happiness (Thompson and Bruk-Lee, 2021). In addition, among a sample of nurses, Vallone and colleagues demonstrated that work demands and resources contribute to their life satisfaction and their degree of physical and psychological diseases (Vallone et al., 2020). Also, Grebner and colleagues found some evidence that the overall well-being of employees can be predicted by their working conditions (Grebner et al., 2005). However, their study relied on a very small sample size that mainly consisted of young workers. Furthermore, in their study general well-being was defined as the absence of psychosomatic complaints and irritated reactions. In this study, we will define general well-being in line with Ed Diener’s definition of subjective well-being (Diener, 1984). According to this definition, subjective well-being is composed of two dimensions: an evaluative dimension and an affective dimension. As such, we define high subjective well-being as a reflection of positive emotions and thoughts about life in terms of frequent positive affect and infrequent negative affect (the affective dimension) and a sense of high satisfaction with life as a whole (the evaluative dimension; Diener, 1984; Diener and Chan, 2011). To conclude, building on these findings and the JD-R theory, the aim of this study is to empirically test a model demonstrating how characteristics of the job contribute to employees’ overall subjective well-being.

### Aim of the study

In the present study, we aim to examine how job characteristics affect employees’ general subjective well-being. To this end, we built a complex model based on the seminal Job-Demands Resources theory (Demerouti et al., 2001; Bakker and Demerouti, 2007; Bakker and Demerouti, 2017) concerned with the well-being of employees. We propose that job demands and resources contribute to employees’ subjective well-being. Based on previous research on the spill-over effect between work-related and general well-being (Bowling et al., 2010; Hakanen and Schaufeli, 2012; Upadyaya et al., 2016; Weziak-Bialowolska et al., 2020; Mazzetti et al., 2023), we furthermore assume that job satisfaction mediates the relationships between demands and resources on the one hand and SWB on the other hand. Building on the JD-R framework and the studies introduced above we thus formulate the following research hypotheses:

*H1*: Leadership is positively related to job satisfaction (H1a). This relationship is mediated by both job demands (H1b) and job resources (H1c). Leadership is negatively related to job demands (H1b) and positively to job resources (H1c).

*H2*: Job demands (H2a) are negatively associated with employees’ job satisfaction whereas job resources (H2b) are positively associated with employees’ job satisfaction.

*H3*: Job demands (H3a) are negatively associated with employees’ overall subjective well-being whereas job resources (H3b) are positively associated with employees’ subjective well-being.

*H4*: Job satisfaction mediates the relationships between job demands and subjective well-being (H4a) and job resources and subjective well-being (H4b).

**Figure 1:**
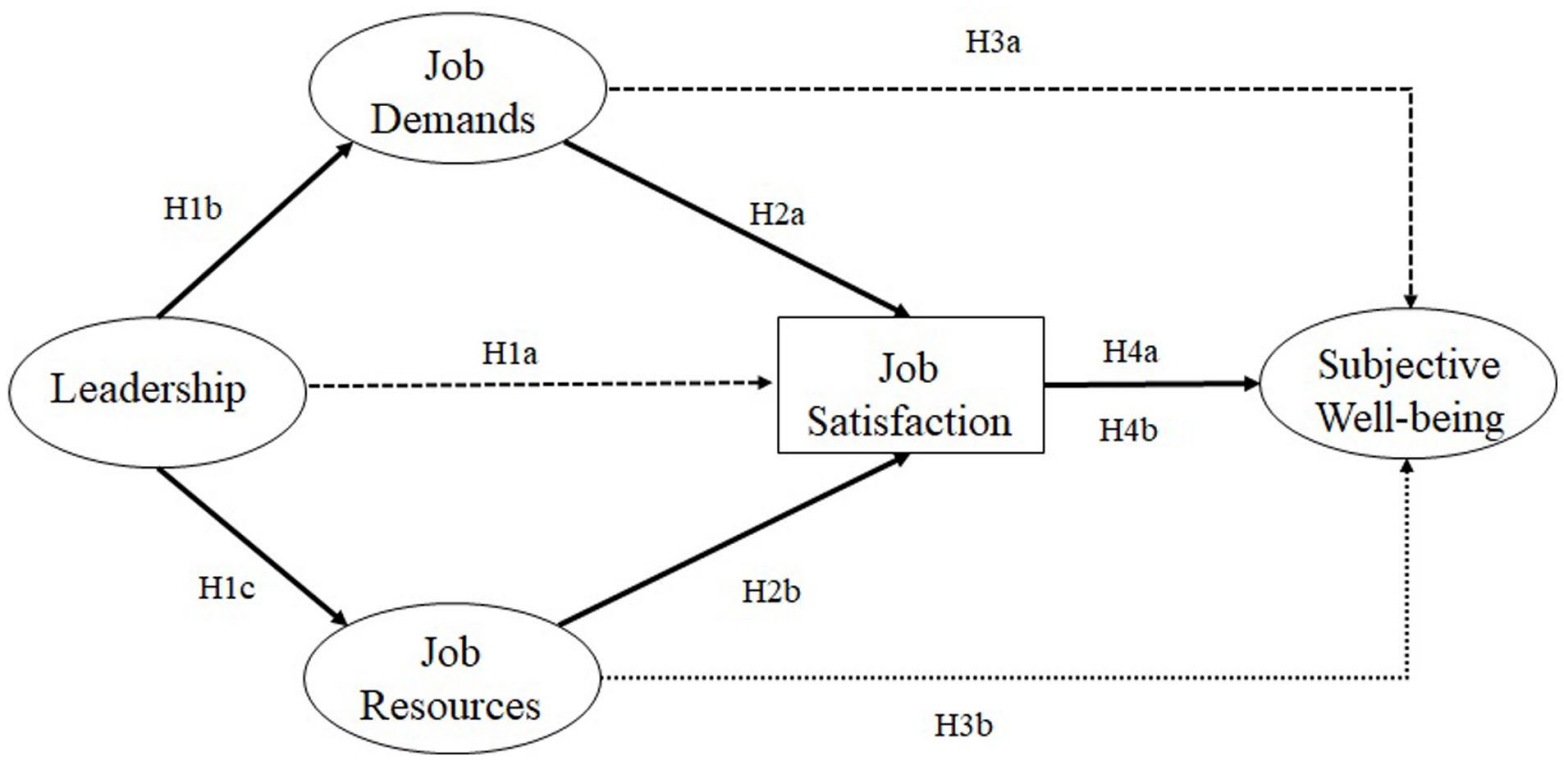
Overview of the conceptual model and study hypotheses.

An overview of our research model and study hypotheses is represented in Figure 1.

Thus, this study builds on past research in three ways. First, attempting to link job demands and job resources directly with employees’ general subjective well-being, we extend current research on the JD-R model that mainly focused on work-related indicators of well-being as outcomes. Second, testing this complex model will also add to research on the spill-over between work-related well-being and overall well-being by demonstrating the role of job characteristics in this relationship. Third, the current study defines general well-being broadly and in line with Ed Diener’s definition (Diener, 1984) of subjective well-being. Previous research on the impact of working conditions on general well-being defined well-being rather narrowly as either the absence of psychosomatic complaints and irritated reactions (Grebner et al., 2005), the single question “How happy are you?” (Thompson and Bruk-Lee, 2021) or life satisfaction and the absence of physical and psychological disease (Vallone et al., 2020).

## Methods

### Data source and sample

The present study uses the cross-sectional data of the second part of Belgian National NN-UGhent happiness study held between February and April (Annemans, 2020). During this period, people completed an online anonymous questionnaire after obtaining approval from the Ethical Committee of the Ghent University Hospital. Inclusion criteria to participate were living in Belgium and sufficient understanding of Dutch or French. More information on the Belgian National NN-UGhent happiness study can be consulted elsewhere (Vandepitte et al., 2022). In total 2,254 people belonging to the Belgian work force completed the questionnaire. However, 395 participants with missing values on our study variables were excluded. The participants’ characteristics are outlined in Table 1.

**Table 1:** Characteristics of the study participants.

	Continuous: mean (SD)	Minimum	Maximum
	Categorical: % (*n*)		
Age, mean (SD)	43.69 (11.26)	18	77
Equivalized income, mean (SD)	€ 2025.37 (1096.14)	€ 0	€ 7,350
**Gender, % (*n*)**			
Male	40.9% (760)		
Female	58.6% (1,096)		
Trans person	0.2% (3)		
**Educational level, % (*n*)**			
Low	6.7% (125)		
Middle	22.9% (426)		
High	70.4% (1,308)		
**Region, % (*n*)**			
Flanders	77.1% (1,433)		
Brussels	6.3% (117)		
Wallonia	16.6% (309)		
**Occupational status, % (*n*)**			
Blue-collar worker	8.9% (166)		
White-collar worker	50.1% (932)		
(Public) functionary	33.8% (629)		
Self-employed	7.1% (132)		

### Measures

#### Outcome variables

##### Subjective well-being

Subjective well-being is composed of life evaluation, positive affect and negative affect. In our model we reversed the score for negative affect, as high subjective well-being, according to Diener’s definition (Diener, 1984) can be understood as a reflection of positive emotions and thoughts about life in terms of frequent positive affect and infrequent negative affect and a positive life evaluation. This definition of subjective well-being is also interpreted as hedonic well-being (Diener et al., 1999; Stephan et al., 2023).

*Life evaluation* (the evaluative component of subjective well-being) was measured with the widely known Cantril Ladder (Cantril, 1966; Bjørnskov, 2010; Veenhoven, 2012) asking respondents to evaluate their life at present time on a ladder from 0 to 10, whereby 0 represents the worst possible life and 10 represents the best possible life.

*Positive and negative affect:* Positive and negative affect (the affective component of subjective well-being) were measured using the Positive and Negative Affect Schedules (PANAS; Watson et al., 1988). For 10 positive and 10 negative emotional states, participants indicated how often they experience each state on a 5-point Likert scale. Positive affect was the sum of the positive emotions items divided by the number of items; correspondingly negative affect was the sum of negative emotions divided by the number of items. Hence, their respective scales ranged from 1 to 5. In the model the score for negative affect was reversed.

##### Job satisfaction

Based on the OECD guidelines on measuring subjective well-being (OECD, 2013), participants rated their job satisfaction on a scale from 0 (totally dissatisfied) to 10 (totally satisfied). Job satisfaction is furthermore considered as an indicator of hedonic work-related well-being (Bakker et al., 2023).

#### Independent variables

##### Leadership

We follow previous studies in their point of view on leadership as an antecedent of job demands and resources as leaders shape the work environment (Schaufeli, 2015; Bakker and Demerouti, 2017; Berthelsen et al., 2018; Tummers and Bakker, 2021). In this study, quality of leadership was determined by employees’ leadership satisfaction and perceived supervisor support. In the leadership literature, various beneficial leadership styles have been proposed that lead to positive outcomes in employees such as transformational leadership (Arnold, 2017), health-promoting leadership (Yao et al., 2021) and more (Tummers and Bakker, 2021). We assume that both employees’ satisfaction with leadership and their perceived supervisor support are the common denominator of such beneficial leadership styles as they all aim to provoke these positive attitudes in employees.

*Supervisor support:* Supervisor support was measured by means of the 4-item Perceived Supervisor Support Scale (Rhoades et al., 2001). Each item was rated on a 5-point Likert scale ranging from 1 (totally disagree) to 5 (totally agree). Perceived supervisor support equals the sum of the items.

*Supervisor satisfaction:* Supervisor satisfaction was assessed on a scale ranging from 0 (totally unsatisfied) to 10 (totally satisfied).

##### Job demands

As job demands, we distinguish role conflict, job insecurity, perceived working conditions and perceived work-private conflict. The included demands are typical hindrance demands, i.e., demands that hinder the employee and are perceived as very unpleasant. As opposed to challenge demands, i.e., demands that can be seen as rewarding work experiences and thus as worth the effort, hindrance demands have the potential to be more detrimental for the well-being of employees (Bakker and Demerouti, 2017).

*Role conflict*: Inspired by Vandenbroeck et al. (2017) we assessed role conflict using a single item (“I get conflicting orders”).

*Job insecurity:* Job insecurity was assessed using 2 items of the four-item Job Insecurity Scale (De Witte, 2000), namely “I feel insecure about the future of my job” and “I think I will lose my job in the near future.” The items were scored on a 5-point Likert scale ranging from 1 (totally disagree) to 5 (totally agree).

*Work-private conflict:* Work-private conflict was measured on a scale ranging from 0 (totally satisfied) to 10 (totally dissatisfied). As such, a higher score represents a greater perceived work-private conflict.

*Perceived working conditions:* On a scale from 0 (totally satisfied) to 10 (totally unsatisfied) participants rated how they perceived their working conditions. As such, a higher score indicates that working conditions are perceived as detrimental.

##### Job resources

As job resources, we distinguish basic psychological need satisfaction at work, skill utilization and personal growth. The choice for these resources can be motivated based on the definition of job resources according to the JD-R model: the aspects of the job that facilitate goal achievement, and stimulate personal growth and development (Demerouti et al., 2001; Bakker and Demerouti, 2017).

*Basic psychological needs at work—autonomy, competence, and relatedness:* The Self-Determination Theory identifies three basic intrinsic psychological needs essential for people to flourish: autonomy (a sense of psychological freedom), competence (sense of effectiveness and mastery) and relatedness (sense of connection with important others; Ryan and Deci, 2000). In this study, these needs were measured with a shortened version of the Basic Psychological Need Satisfaction and Frustration Scale (12 items; Chen et al., 2015) and adapted to the work context. This scale combines a balanced combination of satisfaction (positively formulated) and frustration (negatively formulated) items (Vansteenkiste and Ryan, 2013). A score for each psychological need was calculated by combining a positive and a negative item on a 5-point Likert scale ranging between 0 (never) and 4 (always). Scores for each psychological need were then calculated by subtracting the positive item from the negative item of each particular need resulting in a score between −4 (high frustration) and 4 (high satisfaction).

*Skill utilization:* Skill utilization was measured with a single item (“My work makes sufficient use of all my skills or abilities”). Participants scored this item on a 5-point Likert scale ranging from 1 (almost never) to 5 (always).

*Personal growth:* Inspired by the Work and Meaning Inventory (WAMI; Steger et al., 2012), personal growth (*cf.* the Meaning making through work subscale) was measured using a single item (“I view my work as contributing to my personal growth”). This item was rated on a 5-point Likert scale ranging from 1 (totally disagree) to 5 (totally agree).

#### Control variables

The following socio-demographic variables were included as control variables: age, gender, education level, region, occupational status, and equivalized income (EUROSTAT, 2018; see Table 1).

### Statistical analysis

**Figure 2:**
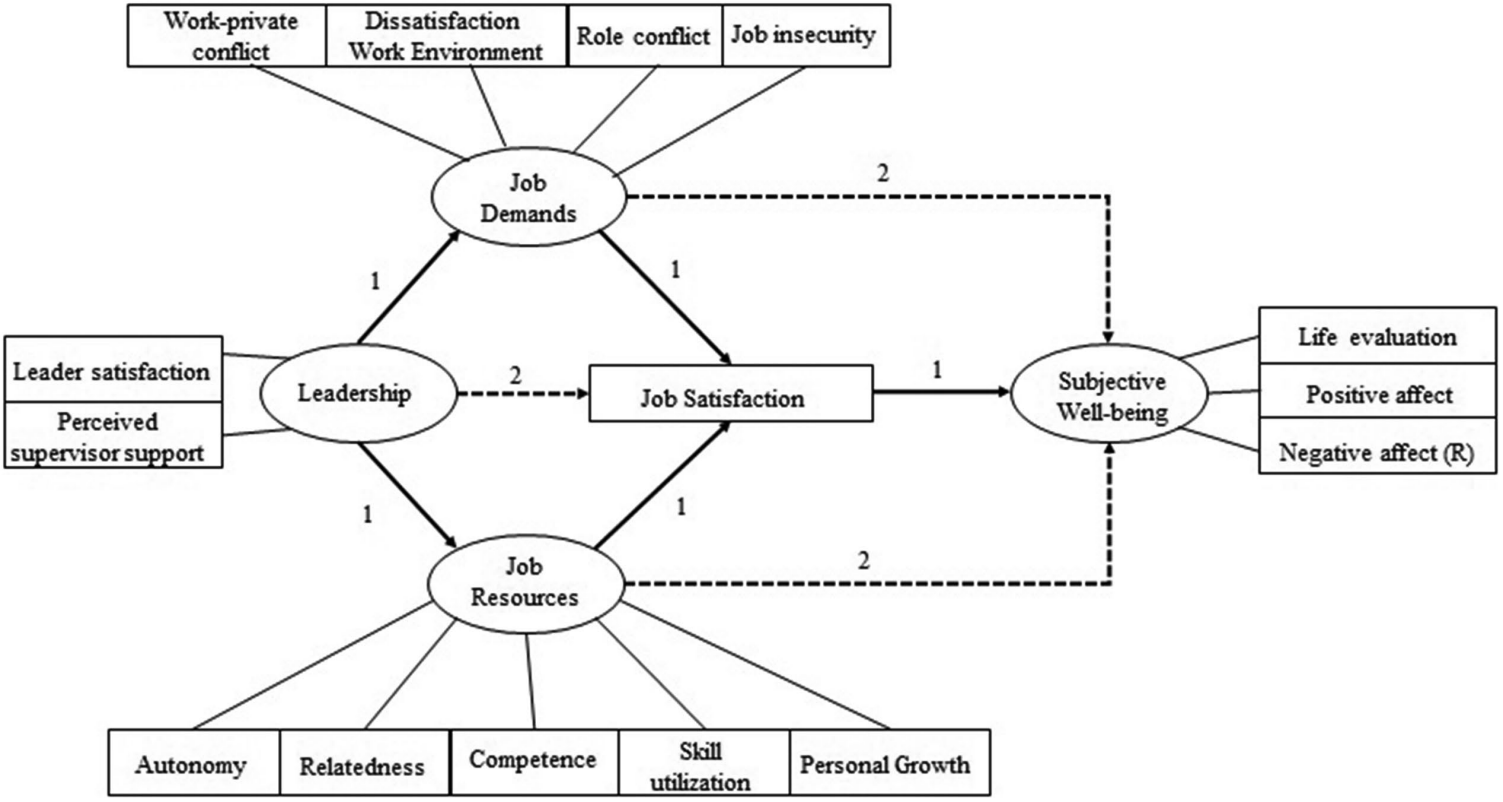
Overview of model 1–2.

Data analyses were conducted using the R package lavaan (Rosseel, 2012). A two-step approach was applied. First, Confirmatory Factor Analyses (CFA’s) were conducted to investigate the pattern by which each indicator loads on the hypothesized factor or latent variable. Second, the relationships between the different constructs was investigated by developing 2 structural models. The first model encompassed paths from leadership to both job demands and job resources, which in turn were related to job satisfaction, and finally job satisfaction was related to subjective well-being (SWB). In the second model, the same pathways as the first model were included, plus a direct path from leadership to job satisfaction, and from both job demands and job resources to SWB. An overview of the two models is illustrated in Figure 2.

Multiple indices were used to examine the overall fit of the hypothesized models. In large samples (as in this study), the χ^2^ test almost always results in the rejection of the model because the difference between the sample covariances and the implied population covariances generates a higher χ^2^ value as the sample size increases (Hu and Bentler, 1999). Therefore, we relied on alternative fit measures. The Root Mean Square Error of Approximation (RMSEA) was used as an absolute goodness-of-fit index. Values below 0.05 for RMSEA indicate good fit, 0.06–0.08 reasonable fit, 0.08–0.10 mediocre fit, and values over 0.10 indicate poor fit (Hu and Bentler, 1999). As relative goodness-of-fit indices the Comparative Fit Index (CFI) and Tucker-Lewis Index (TLI) were evaluated. For these two indices, values over 0.90 indicate good fit (Hu and Bentler, 1999). The fit of nested models was compared by testing the significant changes in the χ^2^ values and by evaluating the Akaike Information Criterion (AIC). Smaller AIC values are indicative for a better fitting model (Akaike, 2011). To test the statistical significance of the mediating and indirect relations bootstrapping procedures were applied. The reported results are based on confidence intervals set at 0.95 with 1,000 resamples.

## Results

### The measurement model

The model consisted of 4 latent variables: leadership, job demands, job resources, and subjective well-being (in terms of life evaluation, positive affect and negative affect); and one observed, single item variable: job satisfaction. After analyzing the modification indices and evaluating the conceptual interrelatedness, the initial model was redefined by allowing error covariance between *Skill utilization* and *Personal growth* and between *Relatedness* and *Competence at Work*. Skill utilization addresses the extent to which employees can make use of their skills, which is naturally related to the degree to which they can improve these skills and experience personal growth. Competence reflects a sense of effectiveness and mastery (Ryan and Deci, 2000). Since the work context is a performance centered environment, it is not surprising that employees who meet these performance standards, have a greater sense of belonging. The values of this redefined measurement model were 0.919 (CFI), 0.892 (TLI), and 0.081 (RSMEA). The CFI value thus indicate a good fit. The RSMEA did not meet its criterion, however, it is still below 0.10. Table 2 shows the results of this final measurement model. The means, standard deviations and correlations of the studied variables can be consulted in Supplementary Table A1.

**Table 2:** Psychometric characteristics of the indicators and the hypothesized higher order factors.

Hypothesized factor	Indicator	Number of items	Factor loading	Cronbach’s alpha
Leadership	Leadership satisfaction	1	0.939	-
	Perceived supervisor support	4	0.862	0.85
Job demands	Role conflict	1	0.433	-
	Work-private conflict	1	0.604	-
	Job insecurity	2	0.421	0.86
	Dissatisfaction work environment	1	0.565	-
Job resources	Autonomy	4	0.854	0.73
	Relatedness	4	0.672	0.74
	Competence	4	0.639	0.72
	Personal growth	1	0.653	-
	Skill utilization	1	0.576	-
Subjective well-being	Life evaluation	1	0.682	-
	Positive affect	10	0.726	0.87
	Negative affect	10	0.671	0.86

### Structural equation models

The first model included paths from leadership to both job demands and job resources, which in turn were related to job satisfaction, and finally job satisfaction was related to subjective well-being (SWB). The second model encompassed the same pathways as the first model plus a direct path from leadership to job satisfaction, and from both job demands and job resources to SWB. In both models age, gender, education level, region, occupational status, and equivalent household income were also included as control variables. Figure 2 offers an overview of the two models.

**Figure 3:**
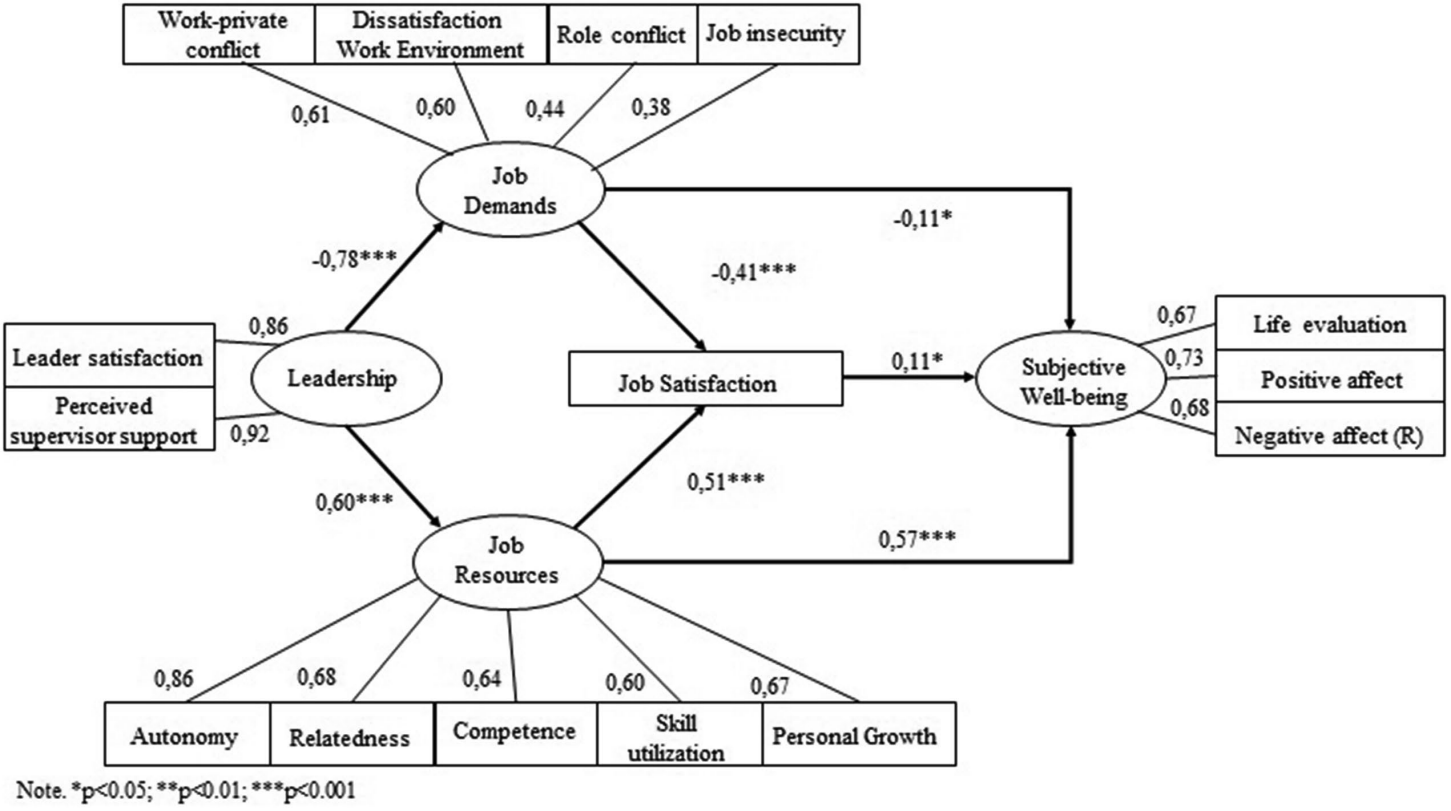
Standardized estimates for the final model (model 3).

Table 3 presents all the model fits. Comparison of the different models based on both AIC measures and the χ^2^ difference test shows that the second model best fitted the data. We subsequently removed the insignificant path from leadership to job satisfaction from this model, creating model 3. The significant paths are shown in Figure 3. The values of the final model were 0.952 (CFI), 0.937 (TLI) and 0.075 (RMSEA), implying a reasonable to good model fit. In addition, a large proportion of the variance in both job satisfaction (R^2^ = 0.611) and subjective well-being (R^2^ = 0.547) is explained by our model.

**Table 3:** Model fit statistics (*n* = 1,859).

Model	Model description	Χ^2^	df	CFI	TLI	RMSEA	Model comparisons	AIC	Δχ^2^	Δdf
Model 1	Fully mediated model	2567.358	207	0.947	0.931	0.078		88762.089		
Model 2	Model 1 plus direct paths from leadership to job satisfaction and from job demands and job resources to SWB	2322.918	204	0.952	0.937	0.075	2 vs.1	88523.650	244.44^***^	3
Model 3	Model 2 without insignificant paths	2323.003	205	0.952	0.937	0.075	4 vs.1	88521.734	244.35^***^	2

^*^p < 0.05; ^**^p < 0.01; ^***^p < 0.001.

Our results support the assumptions of the seminal Job-Demands Resources theory. In the final model, job demands and job resources are both related to job satisfaction. Job resources are positively related to job satisfaction (β = 0.51, *p* < 0.001) whereas job demands are negatively related to job satisfaction (β = −0.41, *p* < 0.001). Leadership was directly related to job demands and job resources. According to our results, leadership was stronger related to job demands (β = −0.78, *p* < 0.001) than to job resources (β = 0.60, *p* < 0.001). In addition, leadership was indirectly associated with job satisfaction through job demands (β = 0.32, *p* < 0.001) and job resources (β = 0.30, *p* < 0.001).

Moreover, our results show that job resources and job demands are both directly related to SWB. Our results also suggest that job resources (β = 0.57, *p* < 0.001) are more strongly related to SWB, compared to job demands (β = −0.11, *p* < 0.001). Moreover, the indirect relations of both job resources (β = 0.05, *p* = 0.032) and job demands (β = −0.04, *p* = 0.044) on SWB were significant. Lastly, the results indicate that work-related well-being is related to general well-being (spill-over effect) as job satisfaction is directly related to SWB (β = 0.10, *p* = 0.032).

## Discussion

This study investigated how the work environment (in terms of job demands and job resources) contributes to employees’ subjective well-being (SWB; in terms of life evaluation, positive affect and negative affect). To this end, we built further on the seminal JD-R model by hypothesizing that the demands and resources in the workplace not only affect well-being at work, but also directly affect SWB. Research (Bowling et al., 2010; Hakanen and Schaufeli, 2012; Upadyaya et al., 2016; Weziak-Bialowolska et al., 2020; Mazzetti et al., 2023) previously illustrated the existence of a spill-over effect from work-related to general well-being but did not investigate how the work environment itself directly contributes to general well-being. The present study further builds on these findings by proposing a complex model that combines leadership, job demands, and job resources as factors contributing either indirectly (via job satisfaction) or directly to employees’ subjective well-being (SWB). Moreover, previous research on the impact of working conditions on general well-being defined well-being rather narrowly as either the absence of psychosomatic complaints and irritated reactions (Grebner et al., 2005), the single question “How happy are you?” (Thompson and Bruk-Lee, 2021) or life satisfaction and the absence of physical and psychological disease (Vallone et al., 2020) while the current study defines general well-being broadly and in line with Ed Diener’s definition of subjective well-being (Diener, 1984).

In order to investigate the hypothesized associations, we built structural equation models. The final model included paths from leadership to both job demands and job resources, which in turn were related to job satisfaction, and finally job satisfaction was related to subjective well-being. Also, direct paths from both job demands and job resources to SWB were included in this final model. The CFI and TLI values were both indicative for a good model fit. The RSMEA, on the other hand, did not meet its criterion, however, it is still below 0.10. This inconsistency should not mean that our model is miss-specified. In their article, Lai and Green further elaborate on explanations for such inconsistency between fit measures (Lai and Green, 2016).

First of all, our results provide further support for the seminal Job-Demands Resources theory. Partially in line with our first hypothesis, job demands and resources strongly mediate the relationship between leadership and job satisfaction, and no direct association between leadership and job satisfaction was found. These results are in line with prior research (Nielsen et al., 2008; Schaufeli, 2015) and indicate that leadership is crucial for establishing employees’ work-related well-being in the way leaders construct the work-environment in terms of job demands and job resources. Leadership appeared to be directly related to both job demands and job resources. This result is not surprising as leaders shape the work environment. Contrary to the results of prior research (Schaufeli, 2015; Berthelsen et al., 2018) however, leadership was stronger related to job demands (β = −0.78, *p* < 0.001) than to job resources (β = 0.59, *p* < 0.001).

Next, and in support of our second hypothesis, job demands and job resources are both related to job satisfaction. Job resources are positively related to job satisfaction whereas job demands are negatively related to job satisfaction. These findings are in line with the fundamental assumptions of the JD-R model (Bakker and Demerouti, 2007; Bakker and Demerouti, 2017; Bakker et al., 2023).

Furthermore, the results of our study now illustrate how job demands and resources contribute to employees’ SWB. Consistent with our third hypothesis, job resources and job demands were indeed *directly* related to SWB. Our results thus further build on those of Grebner et al. (2005) who showed that working conditions are related to psychosomatic complaints and irritated reactions. This finding also corresponds to recent research that showed that job demands are related to employees’ happiness (Thompson and Bruk-Lee, 2021). Our study further added to this research by not only investigating the role of job demands, but also of job resources in predicting SWB. Our results furthermore show that job resources (β = 0.57, *p* < 0.001) are more strongly related to SWB than job demands (β = −0.11, *p* < 0.001). This is line with the findings of a study that demonstrated that demands and resources contribute to nurses’ life satisfaction, physical and psychological diseases (Vallone et al., 2020). In sum, the evidence thus suggests that work (in terms of job demands and job resources) is an important contributor to employees’ SWB.

Lastly, in line with the fourth hypothesis, our results show that job satisfaction is related to SWB (in terms of life evaluation, positive affect and negative affect). This evidence corresponds to the results of prior research that demonstrated a spill-over between work-related well-being and general well-being (Bowling et al., 2010; Hakanen and Schaufeli, 2012; Upadyaya et al., 2016; Mazzetti et al., 2023). Based on prior evidence in combination with the findings of the present study, it becomes clear that well-being at work contributes to employees’ SWB. This study further adds to the literature on the spill-over between work-related and general well-being by demonstrating that job satisfaction does not fully mediate the relationships between job demands and job resources and SWB. As such, the characteristics of the job contribute to employees’ overall SWB beyond the effect of job satisfaction.

### Practical implications

This study aimed to explore how aspects of the job directly and indirectly affect employees’ SWB. The findings of the current study suggest that job demands and job resources contribute both indirectly (via job satisfaction) and directly to SWB. Creating a supportive and healthy work environment is thus of paramount importance in order to foster employees’ SWB. The overall well-being of employees should not be overlooked since it is known that ‘happy’ individuals are more productive, more resilient and have better overall health (Lyubomirsky et al., 2005; Diener and Chan, 2011; Diener et al., 2018). A recent meta-analysis demonstrated that employees’ SWB can be fostered within the workplace (Sakuraya et al., 2020). However, to date, workplace interventions to promote SWB in employees are mainly focused on the individual level (i.e., adapting the individual and their characteristics) and not on the organizational level (i.e., modifying the characteristics of the job and work environment; Sakuraya et al., 2020). Our results seem to demonstrate that organizational level interventions are valuable to foster employees’ SWB. In particular and in line with previous research (Nielsen et al., 2017), our results suggest that investing in increasing job resources such as possibilities for learning and development, supportive relationships with coworkers, and participation in decision making, may be a fruitful approach to promote employees’ SWB. Organizations are also advised to minimize demands where possible as they seem to negatively affect the overall well-being of their employees. Lastly, organizations are well encouraged to cultivate supportive leaders who provide sufficient resources to their employees. Our results indeed highlight the pivotal, although indirect, role of leaders. Leaders make or break the work environment as they directly affect the amount of demands and resources in the workplace. A study of Stansfeld et al. (2015) indeed shows a small benefit from managerial training on employees’ overall well-being. The authors conclude that training of managers might not be sufficient to boost the well-being of employees. Our results seem to support this conclusion as job demands, resources and job satisfaction also contribute to employees’ SWB. As such, we advocate for multicomponent well-being interventions targeting different aspects of the job and the organization and for more research on their effects.

### Strengths and weaknesses

Some strengths and weaknesses need to be mentioned allowing to better understand the context of these findings. A first strength is that the study had a large sample size of people belonging to the Belgian work force (*N* = 1,859). Second, we built on a well-researched model to investigate the impact of job demands and job resources not only on work-related well-being (i.e., job satisfaction) but also on SWB. As such the study contributed to the research field by adding job characteristics as potential direct and indirect contributing factors to SWB. Third, to the best of our knowledge this is the first research on the impact of job characteristics on general well-being that defines general well-being in line with Diener’s definition of subjective well-being (Diener, 1984).

Our study also had some weaknesses. First, although it was definitely a strength that many concepts were questioned, this resulted in the need to slightly adapt several measurements in order to limit the time needed to complete the full questionnaire to avoid response bias. Consequently, several measurements were no longer strictly validated measures and some concepts were questioned using a single item. Nevertheless, the Cronbach’s alpha’s of all shorted scales were sufficient. Also, when Dutch or French versions of the scales were not available in the literature, we had to translate them using a forward back translation method. Second, the cross-sectional nature of the data preclude causal interference. Also, our cross-sectional study design is not ideal for mediation analysis (Stone-Romero and Rosopa, 2008). Nevertheless, our findings are still of value when interpreted correctly. A common misunderstanding about Structural Equation Modeling is that this technique aims to confirm causal relations on the basis of mere associations (Bollen and Pearl, 2013). We made our causal assumptions based on prior research. The observed model fit and results do not “confirm” these assumptions, but rather make them more plausible. Future research employing a longitudinal design is needed to further replicate and support the results of the current study. Third, in order to improve the fit of the CFA models (see Table 2), Modification Indices have been used to allow error covariance. Although, this *post hoc* optimization strategy is generally discouraged, it can be defended in this case due to high conceptual interrelatedness (MacCallum et al., 1992). Fourth, although subjective well-being (Diener, 1984) is a valuable outcome for both organizations (Schulte and Vainio, 2010; Bakker and Oerlemans, 2011) and society (Diener and Chan, 2011; Ngamaba et al., 2017), in this study we only investigated how work contributes to employees hedonic well-being (in terms of job satisfaction and SWB). The well-being literature, however, differentiates between hedonic and eudaimonic well-being (Ryan and Deci, 2001). As such, future research should investigate both types of well-being to better understand how work contributes to employees’ overall well-being in all its facets. Fifth, in accordance with some previous research (Schaufeli, 2015; Bakker and Demerouti, 2017; Berthelsen et al., 2018; Tummers and Bakker, 2021), we considered leadership to be an antecedent of demands and resources at work. However, we assessed leadership as perceived supervisor support and satisfaction with leadership instead of applying measures that assess concrete leadership behaviors. As such, we can draw no conclusions on which specific leadership behaviors contribute to lower demands and higher resources. Inceoglu et al. (2018) identified five types of leadership behaviors that might affect employees’ well-being, namely: change-oriented, task-oriented, relational-oriented, passive and other behaviors. Future research on how these leadership behaviors affect the degree of demands and resources, and employees’ satisfaction with leadership, work-related and general well-being, is warranted. Sixth, all constructs were measured using self-reported measures which raises the question of common method bias. Seventh, since an online questionnaire was used, only people who have access and who are familiar with internet participated. Finally, because people were recruited via social media and a research and consulting company, we have no data on non-response.

It is furthermore important to mention that the outbreak of the COVID-19 pandemic occurred during the data collection. The pandemic and its related lockdown measures greatly impacted the work force as employees were forced to work remotely and even (temporarily) lost their jobs. Hence, pandemic related stress and ambiguity might have affected our results. In addition, telework, at least part time, has now become more common. As such, now more than ever, employees’ well-being might be characterized by the interplay between work and non-work demands and resources (Demerouti and Bakker, 2022). Future research is thus needed to validate our results in a post-pandemic era and to investigate the role of various demands and resources at work and beyond in predicting the well-being of the workforce. A recent study has shown that telework can act as a resource supporting employees’ well-being by buffering the negative effects of high work intensification and low personal resources (Kröner and Müller, 2023). However, more research on the matter is needed to grasp the effects of the new way of working on the well-being of the workforce.

Our results showed that the work environment (in terms of job demands and resources) and job satisfaction contribute to employees’ SWB. However, the demands and resources investigated in this study capture not all aspects of the work environment. Therefore, future researchers should explore the role of other aspects of the work environment (e.g., organizational culture, remote work) in explaining the SWB of employees.

In conclusion, this study contributed to the literature on the JD-R model and SWB by building on the well-researched framework in order to investigate the role of the demands and resources in the workplace on general SWB. Our study indeed showed that job demands and job resources are directly contributing factors to SWB. The study furthermore provided support for the spill-over effect as work-related well-being (i.e., job satisfaction) is related to SWB and mediates the relationships between job demands and resources and SWB. Our results thus suggest that investing in a positive work environment with ample resources is key in supporting employee well-being at work and in life.

## Data availability statement

Upon reasonable request, the raw data supporting the conclusions of this article will be made available by the authors, without undue reservation.

## Ethics statement

The studies involving human participants were reviewed and approved by Ethical Committee of Ghent University Hospital (B670201940146). The patients/participants provided their written informed consent to participate in this study.

## Author contributions

SC: conceptualization, methodology, formal analysis, visualization, and writing—original draft. SV: conceptualization, methodology, and writing—review and editing. EC: writing—review and editing. LA: conceptualization, supervision, and writing—review and editing. All authors contributed to the article and approved the submitted version.

## Funding

This study obtained funding from NN Belgium. The funding resources have no role in the development of the design, conduct, analysis of this study, and final publication decisions.

## Conflict of interest

The authors declare that the research was conducted in the absence of any commercial or financial relationships that could be construed as a potential conflict of interest.

## Publisher’s note

All claims expressed in this article are solely those of the authors and do not necessarily represent those of their affiliated organizations, or those of the publisher, the editors and the reviewers. Any product that may be evaluated in this article, or claim that may be made by its manufacturer, is not guaranteed or endorsed by the publisher.
