# Supplementary material for: How job demands and job resources contribute to our overall subjective well-being
Source: Front Psychol. 2023 Jul 19;14:1220263. doi: 10.3389/fpsyg.2023.1220263 (PMC10394838; doi:10.3389/fpsyg.2023.1220263)
Supplement: Supplementary file 1 [file Table_1.docx]

# Supplementary material

| **Table A1 Descriptive statistics and correlations of the study variables (n=1859)** | | | | | | | | | | | | | | | | | | | |
| --- | --- | --- | --- | --- | --- | --- | --- | --- | --- | --- | --- | --- | --- | --- | --- | --- | --- | --- | --- |
| **Variables** | **M** | **SD** | **1** | **2** | **3** | **4** | **5** | **6** | **7** | **8** | **9** | **10** | **11** | **12** | **13** | **14** | **15** | **16** | **17** |
| **1. Age** | 43.69 | 11.26 | - |  |  |  |  |  |  |  |  |  |  |  |  |  |  |  |  |
| **2. Equivalent income** | 2531.08 | 1028.51 | .09*** | - |  |  |  |  |  |  |  |  |  |  |  |  |  |  |  |
| **3. Leader satisfaction (0-10)** | 6.56 | 2.54 | -.41 | .07** | - |  |  |  |  |  |  |  |  |  |  |  |  |  |  |
| **4. Perceived supervisor support (4-20)** | 13.71 | 3.84 | -.05* | .08** | .81*** | - |  |  |  |  |  |  |  |  |  |  |  |  |  |
| **5. Role conflict (1-5)** | 2.73 | 1.14 | -.07** | .03 | -.30*** | -.30*** | - |  |  |  |  |  |  |  |  |  |  |  |  |
| **6. Job insecurity (1-5)** | 2.10 | 1.05 | -.09*** | .08*** | -.22*** | -.27*** | .25*** | - |  |  |  |  |  |  |  |  |  |  |  |
| **7. Work-private conflict (0-10)** | 3.11 | 2.26 | -.06** | -.03 | -.42*** | -.34*** | .30*** | .20*** | - |  |  |  |  |  |  |  |  |  |  |
| **8. Perceived working conditions (0-10)** | 3.29 | 2.33 | -.07** | -.08*** | -.48*** | -.39*** | .23*** | .14*** | .38*** | - |  |  |  |  |  |  |  |  |  |
| **9. Autonomy (-4 - 4)** | 0.74 | 1.55 | .05* | .12*** | .45*** | .48*** | -.34*** | -.32*** | -.40*** | -.34*** | - |  |  |  |  |  |  |  |  |
| **10. Relatedness (-4 – 4)** | 1.63 | 1.38 | .01 | .14*** | .37*** | .42*** | -.33*** | -.37*** | -.31*** | -.29*** | .58*** | - |  |  |  |  |  |  |  |
| **11. Competence (-4 – 4)** | 1.71 | 1.37 | .18*** | .12*** | .27*** | .28*** | -.34*** | -.36*** | -.31*** | -.24*** | .55*** | .56*** | - |  |  |  |  |  |  |
| **12. Skill utilization (1-5)** | 3.57 | 1.02 | .11*** | .09*** | .31*** | .34*** | -.10*** | -.17*** | -.18*** | -.27*** | .52*** | .33*** | .36*** | - |  |  |  |  |  |
| **13. Personal growth (1-5)** | 3.64 | 1.05 | -.04 | .14*** | .39*** | .42*** | -.09*** | -.16*** | -.28*** | -.28*** | .59*** | .38*** | .34*** | .55*** | - |  |  |  |  |
| **14. Job satisfaction (0-10)** | 6.73 | 2.27 | -.01 | .10*** | .57*** | .50*** | -.29*** | -.36*** | -.46*** | -.40*** | .62*** | .49*** | .42*** | .46*** | .53*** | - |  |  |  |
| **15. Life evaluation (0-10)** | 6.94 | 1.66 | .03 | .16*** | .33*** | .28*** | -.17*** | -.22*** | -.40*** | -.29*** | .41*** | .38*** | .36*** | .23*** | .28*** | .48*** | - |  |  |
| **16. Positive affect (1-5)** | 3.61 | 0.52 | .14*** | .13*** | .24*** | .24*** | -.14*** | -.22*** | -.26*** | -.23*** | .42*** | .42*** | .48*** | .31*** | .35*** | .41*** | .48*** | - |  |
| **17. Negative affect (1-5)** | 2.59 | 0.57 | -.11*** | -.13*** | -.25*** | -.23*** | .23*** | .28*** | .29*** | .23*** | -.38*** | -.40*** | -.47*** | -.23*** | -.26*** | -.34*** | -.45*** | -.52*** | - |
| *p<0.05  **p<0.01  ***p<0.001 | | | | | | | | | | | | | | | | | | | |
